# Exploring Avenues of Predicting Drug-Induced Liver Injury

**DOI:** 10.1021/acs.chemrestox.6c00145

**Published:** 2026-08-17

**Authors:** Don Pivithuru Liyanarachchi

**Affiliations:** 14716 University of Missouri Department of Chemistry, 125 Chemistry Building Columbia, Missouri 65211, United States

## Abstract

Drug induced liver injury prediction is advancing through mechanistic modeling, patient-derived systems, and emerging immune-based biomarkers. Integrating these approaches improves drug safety and reduces development failures.

## Graphical abstract

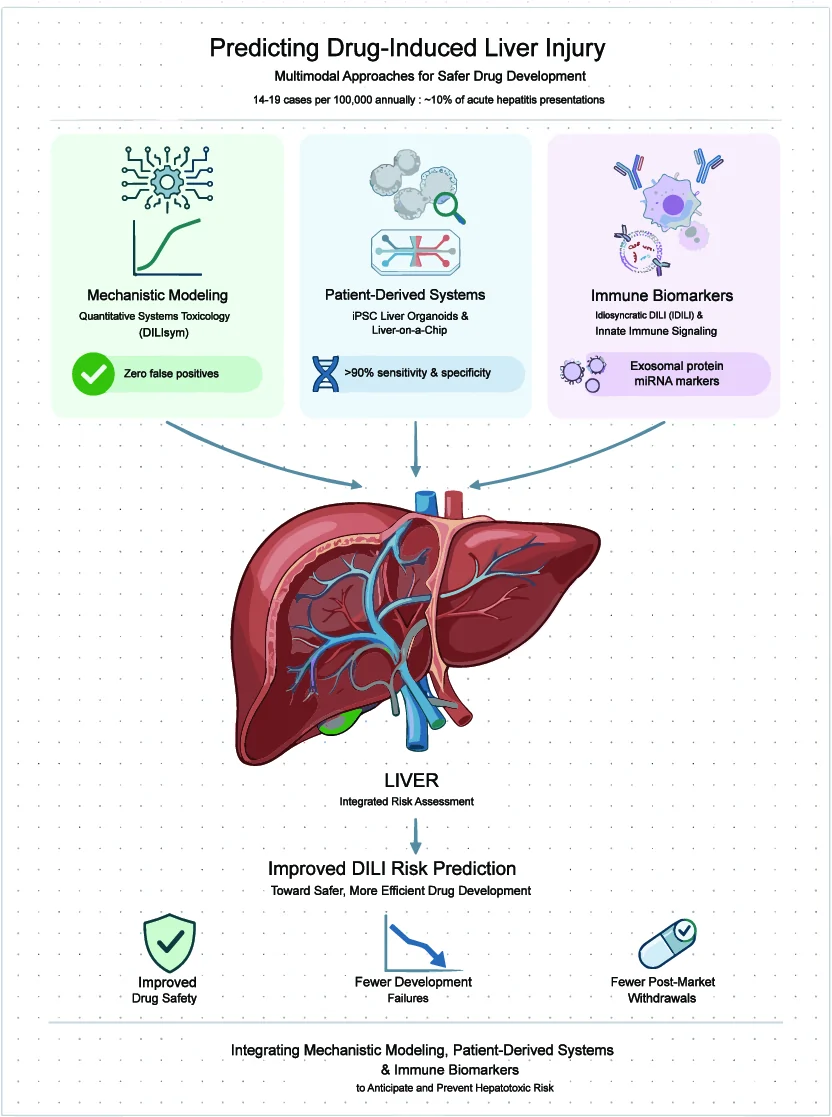

Drug-induced liver injury (DILI) is a major cause of acute liver dysfunction and remains one of the most challenging adverse drug reactions encountered in clinical practice. Epidemiologic studies estimate an annual incidence of 14–19 cases per 100,000 individuals, with DILI accounting for approximately 10% of acute hepatitis presentations and a substantial proportion of acute liver failure in Western countries.^1,2^ Clinical manifestations range from asymptomatic elevations in aminotransferases to fulminant hepatic failure, and the unpredictable nature of idiosyncratic reactions continues to complicate diagnosis and risk assessment.^1^ The severity and continually evolving nature of DILI has pushed the field toward developing predictive approaches that can improve patient safety, avoid late-stage development failures, and prevent costly postmarket withdrawals. This theme was highlighted at the “Predicting Drug-Induced Liver Injury” symposium at the ACS Fall 2025 conference, where the discussions centered on how traditional in vivo and in vitro toxicology methods can be integrated with modern in silico tools. Together, these complementary strategies underscore the growing emphasis on building more reliable and mechanistically informed DILI prediction frameworks.

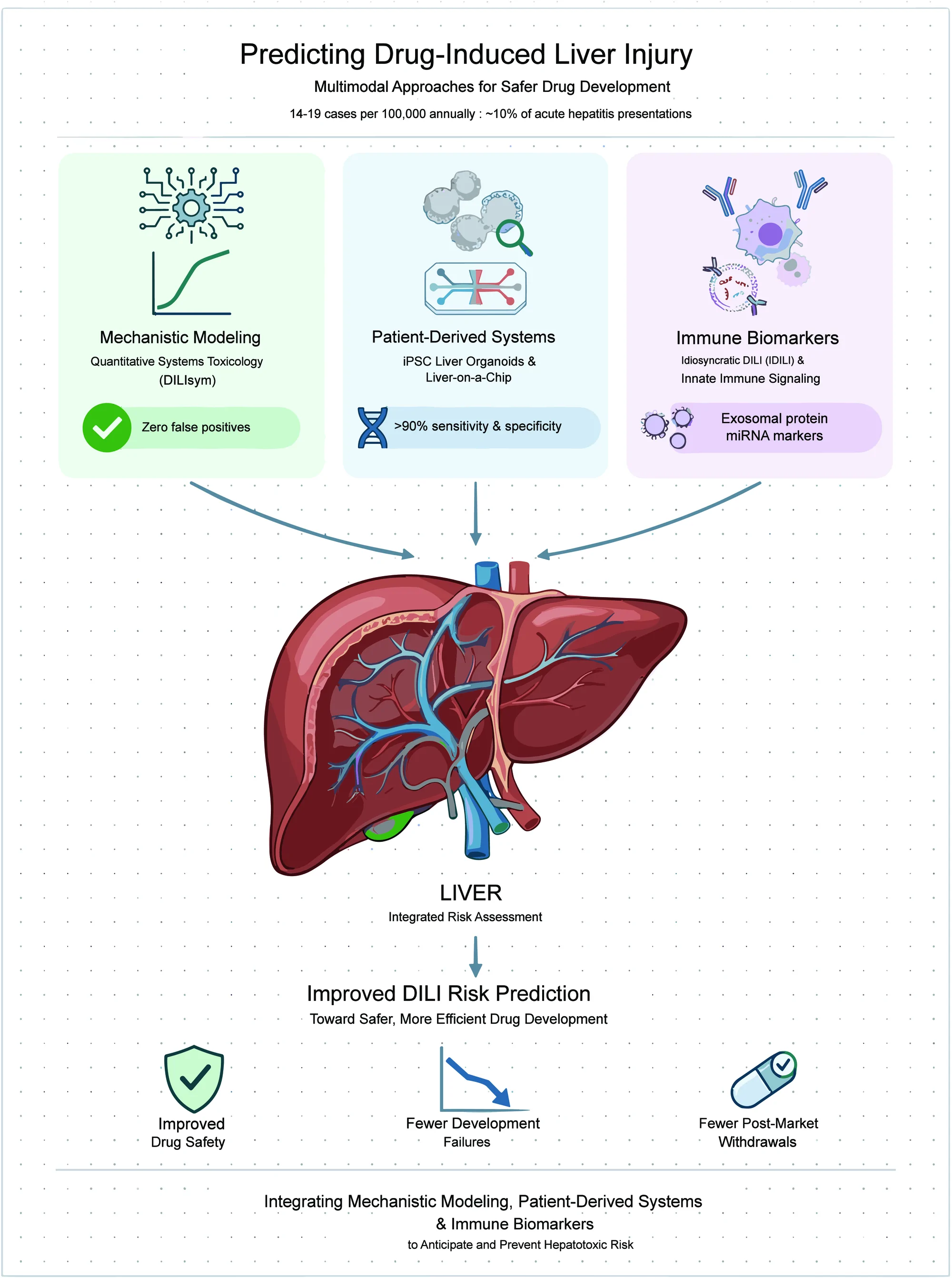



Quantitative Systems Technology (QST) is a mechanistic, computational approach that integrates toxicology, physiology, and pharmacology to predict how drugs cause adverse effects in specific organs.^3^ It uses mathematical models to connect drug exposure with downstream biological disruptions, enabling early, quantitative assessment of safety risks during drug development.^3^ One of the highlights of the ACS symposium was a model of QST that specializes in the prediction of DILI named “DILIsym”. In two talks presented by Paul Watkins (also on behalf of Kyunghee Yang), a use case for DILIsym was outlined in the evaluation of hepatic safety for five calcitonin gene-related peptide inhibitors used in migraine treatment. The DILIsym model assesses compounds across four key mechanisms of hepatotoxicity: mitochondrial dysfunction, reactive oxygen species generation, bile salt export pump (BSEP) inhibition, and multidrug resistance protein 3 (MDR3) inhibition. It generates DILI-risk outputs for each mechanism while also incorporating multiple exposure levels. Using these mechanistic inputs within its evaluator framework, DILIsym predicted the liver enzyme elevations associated with Telcagepant, a first-generation calcitonin gene-related peptide inhibitor, in a manner consistent with the clinically observed DILI risk of the compound. DILIsym also predicted that the four next generation drugs would have a significantly less DILI risk in comparison to Telcagepant, later confirmed through clinical trials. DILIsym has supported drug programs where liver risk was recognized early, such as fezolinetant, and has also helped explain why some first-generation candidates, including small-molecule CGRP inhibitors, were stopped after clinical toxicity. To date, every drug or regimen the model has flagged as a liver safety concern has shown real risk, so there have been no false positives. The main limitation is that roughly one-fifth of drugs known to cause liver injury in some patients, such as ketoconazole, are not detected. These misses likely stem from toxic metabolites that are not formed in the liver cell systems used for testing, and more metabolically capable cultures have not yet corrected this gap. Some injury pathways, including those involving ER stress, also appear to be missing from current assays and from the model itself.

Another talk presented by Jonathan Sexton focused on the merging of in-silico methods with preclinical biological models. Drug-induced liver injury (DILI) remains difficult to predict because standard preclinical systems often fail to capture human-relevant toxicity. An integrated platform combining high-content imaging-based morphologic profiling with Liver-on-a-Chip systems built from iPSC-derived liver organoids (generated by reprogramming adult cells into induced pluripotent stem cells, then differentiating them into functional hepatocytes^4^) from DILI patients improves both prediction and mechanistic insight. The assays target the major mechanistic pathways mentioned before to quantify hepatotoxic risk. Organoids from six genetically distinct patients were screened against a library of known hepatotoxicants and nontoxic controls, with evaluation based on viability, morphology, and stress markers. The platform achieved sensitivity and specificity above 90%, exceeded the performance of conventional in vitro assays, and revealed substantial interindividual variability. It reproduced established mechanisms of toxicity and uncovered patient-specific response signatures. These findings position patient-derived liver organoids as a high-fidelity model for DILI risk assessment and mechanistic study, enabling more accurate safety evaluation and supporting efforts to identify individuals with heightened genetic susceptibility.

A form of DILI dubbed Idiosyncratic drug-induced liver injury (iDILI) is a rare, unpredictable hepatotoxic response that occurs in only a small subset of individuals at normal drug doses. It reflects patient-specific interactions among genetics, reactive metabolites, and immune activation, shows variable latency, and can mimic other liver diseases, making diagnosis difficult and the clinical impact significant.^5^ Jack Uetrecht presented a talk emphasizing possible biomarkers that could be used in the prediction of iDILI. Multiple observations, including CD8 positive T cell infiltration, strong HLA associations, and clinical features resembling viral hepatitis, indicate that hepatocyte loss results primarily from immune driven injury rather than direct drug toxicity. Despite this, most assays focus on cytotoxicity, mitochondrial stress, or BSEP inhibition, none of which reliably capture iDILI risk. Models capable of detecting adaptive immune involvement, such as PD-1 knockout systems, provide useful insight but are not practical for routine screening. Initiation of adaptive immunity requires an innate immune response, which should be consistent across individuals and therefore suitable for biomarker discovery. Several iDILI associated drugs rapidly alter hepatic mRNA signatures consistent with stress and innate immune activation, and macrophages can be activated either by reactive metabolites or by factors released from hepatocytes. These hepatocyte derived components, likely carried in exosomes as proteins or microRNAs, represent promising biomarkers for iDILI risk and could significantly improve drug development.

DILI prediction is advancing through mechanistic modeling, patient-derived systems, and emerging immune-based biomarkers, collectively strengthening our ability to anticipate hepatotoxic risk. Integrating these complementary approaches will be essential for improving drug safety, reducing development failures, and refining our understanding of the diverse pathways that drive both intrinsic and idiosyncratic liver injury.
